# Translation and cross-cultural adaptation of EAT-26 questionnaire in Urdu

**DOI:** 10.1016/j.mex.2023.102343

**Published:** 2023-08-23

**Authors:** Anam Jamil, Nida Zahid, Momina Imtiaz, Hadia binte Obaid, Asma Muhammad, Aniqa Jamil

**Affiliations:** a Department of Rehabilitation Sciences, Shifa Tameer e Millat University, Park road, Islamabad, Pakistan; b Primal Support, E 11/4, Islamabad, Pakistan; c Human Development Research Foundation, Islamabad, Pakistan

**Keywords:** Cultural adaptation, EAT-26 questionnaire, Eating disorders, translation, Translation and cross-cultural adaptation in Urdu

## Abstract

EAT-26 questionnaire is used globally to screen people for the risk of eating disorders. In addition to this, the EAT-26 is being used in its original English version in research and clinical settings due to unavailability of the Urdu version. Therefore, the aim of this study was to introduce the Urdu version of EAT-26 to clinicians and academicians in Pakistan, interested in the assessment of population at risk of eating disorders. After getting the formal permission for translation by Dr. D. M. Garner, WHO guidelines were followed for the translation and adaptation process. Two independent translators with psychological background worked under the supervision of a lead to produce the definitive version following six steps of translation and adaptation. Cognitive interviews and focused group discussions helped in the assessment process for the understanding level of translated Urdu version. The pre-final version showed comprehension and acceptability during initial pilot testing.

• The final translated version of EAT-26 in Urdu will be available on Internet to use. It is expected that the use of EAT-26 will be widespread in Pakistan, aiming at the assessment of eating disorders.
• The Urdu version of EAT-26 is finalized, and ready to use by researchers and clinicians in Pakistan.

## Graphical abstract

**Figure:**
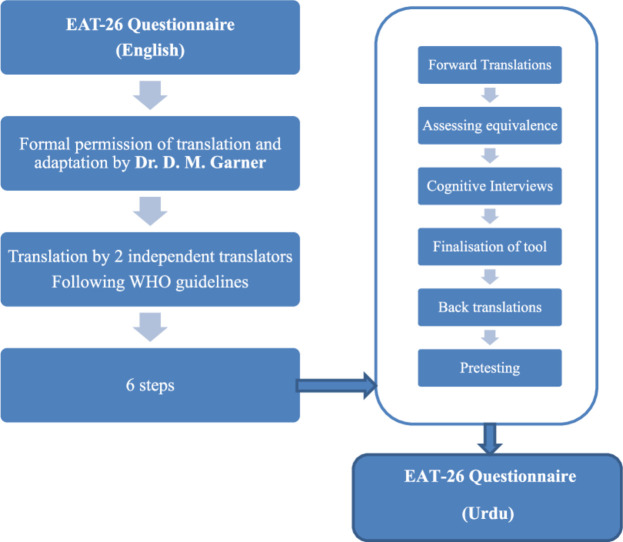

Specifications table

**Table:** 

Subject area:	Psychology
More specific subject area:	*EAT-26 questionnaire*
Name of your method:	*Translation and cross-cultural adaptation in Urdu*
Name and reference of original method:	*N/A*
Resource availability:	*N/A*

Method details

## Study design

Normal growth and development is dependent upon adequate intake of food [1,2]. Eating disorders not only cause medical co-morbidities but also impose psychosocial burden [3]. Lower regional brain volumes and cortical thickness is correlated with eating disorders caused by a sudden increase in weight and negative eating habits [4]. The major classifications of Feeding and Eating Disorders (FEDs) include Anorexia Nervosa (AN), Bulimia Nervosa (BN), Binge Eating Disorder (BED) and Avoidant/Restrictive Food Intake Disorder (ARFID) [5] These disorders are specified by extreme weight-control behaviors including self-starvation due to the fear of becoming fat, or may include binge eating habits followed by vomiting to maintain desired body weight [3,5].

Use of laxatives and misuse of diuretics are the two common compensatory behaviors to lose weight gained through purging and binge eating [3]. In females, body image concerns are of paramount importance. Whereas, women and young girls have a higher prevalence rate of eating disorders including Anorexia Nervosa and Bulimia Nervosa while Binge Eating Disorder is more common in men [5]. Women are three times more likely to suffer with eating disorders as compared to men.

1 in every 100–200 girls gets affected by Anorexia Nervosa whereas 3% of adolescent and young women are affected by Bulimia Nervosa in Western countries but there is dearth of empirical information regarding prevalence rates of eating disorders in low-middle income countries including Pakistan [7]. Due to industrialization, urbanization and globalization, it has been suggested that eating disorders can be more prevalent among high risk populations of third world countries [3]

Various assessment measures are used clinically to assess the eating disorders and its impact on someone's attitude, feelings, and behaviors [6,7]. For this purpose, Eating Attitudes Test (EAT); an eating disorder screening tool was developed by Garner and Garfinkel in 1979 that was later modified to the EAT-26. The version with 40-items with a validity coefficient of 0.87 (*p*<.001) and high internal reliability was organized into five criterion group including anorexics, normal females, normal males, obese females, and recovered anorexics [6]. It was initially used to assess the symptoms of anorexia nervosa [8], which was then further revised after the deletion of fourteen items. EAT-26 is a précised version of EAT-40 as fourteen of the original items did not fit into any of the three factors determined by EAT-40 which include dieting, bulimia and food preoccupation, and oral control [6] EAT-26 is a reliable and valid self-report able, easily administered questionnaire that has been used to distinguish various levels and types of eating disturbances [8]. This screening measure is having high sensitivity and specificity related to being at risk of eating disorders. However, diagnosis of an eating disorder cannot simply be made by using EAT-26 alone. Low percentage of false positivity was observed when applied in a non-clinical population as a screening method for Anorexia Nervosa and Bulimia Nervosa [9].

Various translations have been made in different languages which are used in different countries by clinicians but in Pakistan it is still being utilized in English version that is a major hindrance in screening of eating disorders locally. As the native language of Pakistan is Urdu, and English is not understood by majority of the population [8]. Therefore, there is a dire need of adaptation and translation of many questionnaires including EAT-26 that are developed in Western countries but not adapted for the use in LMICs including Pakistan.

## Methodology

EAT-26 is most widely used valid and reliable assessment measure for eating related disorders. The original EAT questionnaire was developed by consensus panel of National Institute of Mental Health in 1979. This questionnaire consisted of 40 items for the screening of anorexia related symptoms. In 1986, Garner and his colleagues modified and reduced this original version after a factor analysis to create an abbreviated 26-item test; EAT-26 [10]. This questionnaire is used globally to meet the screening need of eating related health concerns. In order to have an assessment tool in Urdu language to be used with Pakistani people, original version of EAT-26 in English was adapted and translated into Urdu. The EAT-26 has been reproduced, translated and adapted with the permission from Dr. David M. Garner.

### Study participants

Participants for cultural adaptation were purposefully selected to represent diverse socio economic status with focus on adolescent girls from the different backgrounds in the community. Female clinicians were also interviewed to have a broader understanding of the local customs and terms used in daily communication.

### Translation process

**Fig. 1:**
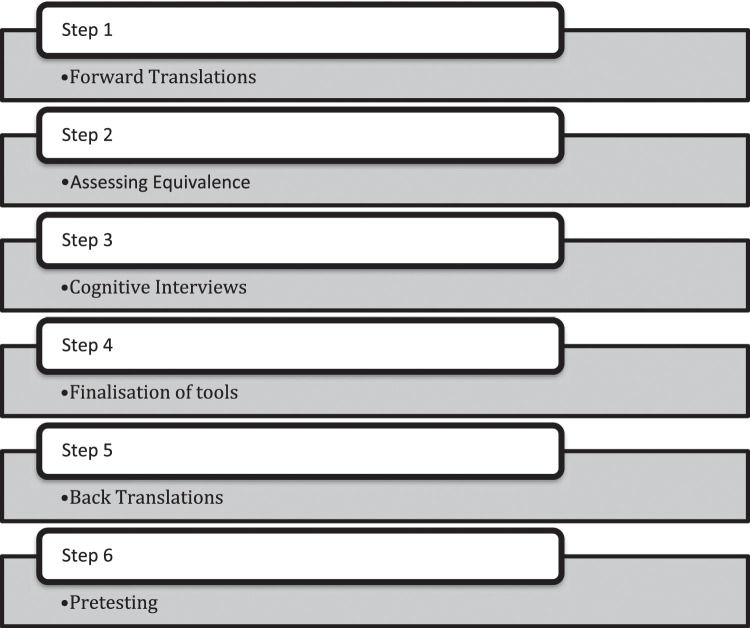
Process of cultural adaptation of EAT-26 in Pakistan.

After getting translation permission, the EAT-26 has been reproduced with the permission by Garner. The translation and cross cultural adaptation of EAT-26 was based on the WHO guidelines for translation and adaptation. According to these guidelines, the whole process consists of basic VI phases. (Fig. 1)

#### Step 1: Forward translation

During the first phase of forward translation, original English version of EAT-26 questionnaire was translated to Urdu by two independent Urdu speaking translators. Two forward translations were made of the questionnaire from English into Urdu. Both professional translators with Urdu as their native language and also fluent in English were selected for this first step. Bilingual translators independently produced the two independent forward translations of the original instructions, statements and response choices.

#### Step 2: Assessing and establishing equivalence

A pooled version of translation was produced by the consensus of both the translators and the local coordinator. This aim was to have a version that is conceptually equivalent and easy to understand by the target population. This was carried out in consultation with developmental experts who checked for conceptual, content, semantic and technical equivalence. Detailed cultural adaptation grids were maintained to record the suggested modifications that helped develop the versions that were then field-tested.

#### Step 3: Cognitive interviews

The adaptation team was trained to conduct cognitive interviews in field and clinic to assess the understanding level of instruments. A combination of techniques was used during cognitive interviews. ‘Paraphrasing’, allowed participants to rephrase the item meaning in their own words [11,12]. “Debriefing” [12] encouraged participants to describe those items that were difficult to comprehend. ‘Probing’ was used to inquire about clarity of instructions and what each item and their response categories meant [11,12]. As per guidelines, a cognitive interview guide was prepared including probes such as, “Can you explain what you have understood by this question?” “What term or phrase will you rather use to explain this item?” “How did you feel when I asked this question?” “How difficult was it for you to answer this question? (Table 1).

**Table 1:** Participants and number of cognitive interviews.

Study site	Cognitive interviews	Participants
Pakistan	3 FGDS	■ 2 with university going girls, two groups (both native Urdu speakers) and other mixed (Potohari& Urdu speaker) (*n* = 12; 4 in each group)
	1 FGDS	■ 1 with female clinician (*n* = 2)

#### Step 4: Finalization of tools

Findings from each cognitive interview resulted in the preparation of technical modification grids, which incorporated field findings, agreements, disagreements and suggested modifications regarding the precision of the translation. These were discussed with the expert panel. Modified version of EAT-26 was then taken back to the next target group in the field for further inputs. This process of cognitive interviewing was repeated for all target groups until no new findings emerged and the participants were clear on all items of each instrument.

#### Step 5: Backward translation

Independent bilingual consultant with a background in Public Health back translated the Urdu version of the EAT-26 into English. It was made sure that the details including name of the questionnaire wasn't shared with the back-translator. Back translation focused on concept and conceptual equivalence and not on linguistic equivalence. A final tool was prepared for pretesting. Comparison of the backward version was performed by the team lead in order to detect any misunderstandings, mistranslations or inaccuracies in the revised version of the questionnaire.

#### Step 6: Pretesting

The final translated questionnaire was presented for pre-testing to 5 adolescent girls, 3 from universities and two from clinic. The focus of pre-testing was to assess appropriateness, understanding and administration time.

A final version of EAT-26 in Urdu was formed after proofreading to avoid typing and spelling errors in Urdu.

## Results

The cultural adaptation and whole translation procedure using the described procedure took six to eight months.

### Forward translations

The primary aim for the translators was to maintain semantic equivalence by paying attention to subtle differences. For example in Urdu, ‘Usually’ was initially translated as “aksar” which on back translation meant ‘often’ and hence was not semantically accurate. This was then replaced by more appropriate term, such as, “aam tor per”, which was a more accurate translation. Another response category, ‘rarely’ was translated as “bohat kum” rather than “ba-mushkil”.

### Cognitive interviews (Field testing)

Cognitive interviewing with the target group i.e. adolescent girls helped in achieving different aims. This helped in an exploration of everyday language which they were accustomed to. Involving local language assessors for assessment purpose and adolescent girls as interviewees particularly for the EAT-26 helped in evaluating the cultural appropriateness of words used for eating habits and routines. Based on the findings from the cognitive interviews, the adaptation team and expert panel made modifications for cultural relevance. Examples of some of the modifications are described below:

### Rephrasing

• EAT-26 item number 12, “Think about burning up calories when I exercise”: The initial translation for ‘burning calories’ in Urdu was “hararay jalana”.Many participants found difficulty understanding it and considered this a vague term leading to an immediate negative response to the item. Probing the item and describing what we were investigating, made participants realised that this behavior is common among them and is being opted by a lot of them. The final modification of the phrase ‘burning calories’ was more acceptable as “hararay kum kerna”, further maintaining conceptual equivalence.
• Similarly in EAT-26 item no 17, “Eat diet foods”: The initial translation for ‘diet foods’ in Urdu was “parhaizi khana”. Through cognitive interviewing, participants reported that the term ‘parhaizi khana’ is somehow linked to the diet of ill people. Therefore, after panel discussion, term “kum hararon wali ghiza” was finalized.

### Back translation

It would be worth mentioning here that, overall, there were no significant differences between the back translated versions and the original English instruments.

### Pretesting

This stage aimed at understanding the practical issues for a clinical based assessment. For example, understanding the average time taken to complete each assessment (EAT-26: 12 - 15 min). This exercise of notifying an average assessment time to the participants was important during the process of informed consent to create clear operational guidelines for the clinical assessment.

## Discussion

EAT-26 is widely used as a valid and reliable screening instrument by clinicians for the assessment for eating disorders. Both, anorexia nervosa and bulimia nervosa are categorized as important eating disorders prevalent among adolescent girls. In Pakistan, the prevalence of eating disorders is somehow related with anxiety, depression and body shape. Most of the cases go unreported because of lack of awareness or fear of getting stigmatized by the society. Instead obesity is being focused to a greater extent, receiving both medical and mental health interventions. Literature suggests that most of such cases are being reported to gastroenterologists with the complaints of experiencing acidity, burning, and indigestion while some cases are also reported to a dentist of having tooth decay, loss of dental enamel, or calcium deficiency in teeth due to binge eating or purging behaviors. Hence, the actual number of individuals with eating disorders is far higher than the number of reported cases in hospitals and other clinical settings.

Furthermore, baseline data regarding prevalence of eating disorders is missing in LMICs including Pakistan. Just a few studies that have been performed in this regard which exhibit some surprising results, including a study performed on nursing college students. This study shows a prevalence of eating disorders among 39.5% nursing college students. For past few decades, eating assessment measures including EAT-26 are being used widely for clinical and research purposes, which indicates the importance of this measure. Many outcome measures including EAT-26 that are developed in Western countries have not been adapted and translated in low middle income countries (LMICs) including Pakistan. Therefore, this study focuses on translation and adaptation of EAT-26 in Urdu to be implemented with Pakistani population. This may help an easy assessment procedure of eating disorders in clinical as well as field settings. Taking this into account, one of the main objectives of the present study was to translate and adapt the EAT-26 to Urdu to be used in rural settings following the WHO guidelines for the translation, and cross-cultural adaptation.

The basic purpose to adapt and translate these instruments was to further use them for research purposes. This study contributed to the adaptation and translation of a valid and reliable instrument following the best evidence-based guidelines of WHO for the translation, and cross-cultural adaptation for its use in two low middle income countries including Pakistan.

In order to ensure consistency, a systematic approach was employed for qualitative adaptation of these tools, to satisfy multiple equivalence needs of an adapted version of instrument among Pakistan. For maintenance of equivalences and to get culture specific version of EAT-26, assessors were trained to administer EAT-26 with equality. Extensive training was provided to all assessors, as a result of which high inter-rater reliability was achieved, though the translation of EAT-26 to Urdu required quite minimum cross-cultural adaptation. Previously some other studies were performed to adapt outcome measures for use in community settings. In Spain, EAT-26 was adapted for better screening of eating disorders among females recruited from community settings. This study demonstrated the need for cultural adaption of items to ensure appropriate outcomes.

In EAT-26 adaptation, few culture based variations were identified to be adapted for its use in Pakistan. For the adaptation of EAT-26, testing materials were reviewed in terms of cultural relevance, and eventually substitutions were identified, discussed, and agreed on in a larger study team with experts for its use in clinical and community settings of Pakistan.

Psychometrics was not the prime focus of our study. The current study aimed to translate and adapt EAT-26 and achieved very good Inter rater reliabilities. If EAT-26 has to be used for clinical and research purpose in Pakistan, it is recommended that future studies are conducted to establish its reliability and validity among Pakistani women.

Future studies using the translated and adapted version of EAT-26 can help to assess psychometric norms further and contribute to the research being taken in clinical and field settings, therefore, helping researchers and health professionals to better identify young women at the risk of eating disorders.

## Declaration of Competing Interest

The authors declare that they have no known competing financial interests or personal relationships that could have appeared to influence the work reported in this paper.

## Data Availability

Data will be made available on request.

## Appendix Supplementary materials

Image, application 1
