# Supplementary material for: Translation and cross-cultural adaptation of EAT-26 questionnaire in Urdu
Source: MethodsX. 2023 Aug 23;11:102343. doi: 10.1016/j.mex.2023.102343 (PMC10472286; doi:10.1016/j.mex.2023.102343)
Supplement: Supplementary file 1 [file mmc1.docx]

**EAT-26 questionnaire – Urdu Version**

**ہدایات:** یہ سوالنامہ آپکی غذائی بد نظمی اخذ کرنے کے لیے ترتیب دیا گیا ہے کہ آپکو ماہرانہ توجہ کی ضرورت ہے کہ نہیں۔ یہ سوالنامہ کسی غذائی مرض کی تشخیص نہیں کرتا اور نہ ہی کسی ماہرانہ مشورے کا نعم البدل ہے۔ براہے مہربانی نیچے دیا گیا سوالنامہ جتنا ممکن ہو سکے اتنی درستگی ،اور دیانتداری سے مکمل پُر کریں۔ اس میں کوئی جواب غلط یا درست نہیں ہے۔ آپکے دیئے گئے تمام جوابات کو صیغہ راز میں رکھا جائے گا۔

**حصہ اول:** مندرجہ ذیل سوالات کو مکمل کریں۔

تاریخ پیدائش: مہینہ: _______ دن: _______ سال: _______

جنس: مرد: _______ عورت: _______

قد: فٹ: _______ انچز: _______

موجودہ وزن: _______ زیادہ سے زیادہ وزن (حمل کے علاوہ): ______________

بلوغت میں کم سے کم وزن: _________

مثالی وزن: _________

**ہدایات:**

| **حصہ دو**ئم: مندرجہ ذیل جملوں میں سے درست جواب پر (✓) کا نشان لگائیے۔ | | **ہمیشہ** | **عام طور پر** | **اکثر** | **بعض اوقات** | **بہت کم** | **کبھی نہیں** |
| --- | --- | --- | --- | --- | --- | --- | --- |
| 1 | مجھے وزن بڑھنے کا خوف رہتا ہے۔ |  |  |  |  |  |  |
| 2 | بھوک ہونے کا با وجود میں کھاناکھانے سے اجتناب کرتا/کرتی ہوں۔ |  |  |  |  |  |  |
| 3 | میں ہر وقت کھانے کی سوچ میں کھویا رہتا /کھوئی رہتی ہوں۔ |  |  |  |  |  |  |
| 4 | جب بھی مجھ پر زیادہ کھانے کا جنون سوار ہوتا ہے تو میں محسوس کرتا /کرتی ہوں کہ شاید میں خود کو روک نہ پاؤں۔ |  |  |  |  |  |  |
| 5 | میں اپنے کھانے کی چیز کے چھوٹے چھوٹے ٹکڑے کرتا /کرتی ہوں۔ |  |  |  |  |  |  |
| 6 | جو کھانا کھاتا /کھاتی ہوں مجھے اُس میں حراروں(Calories) کی مقدار کا علم ہوتا ہے۔ |  |  |  |  |  |  |
| 7 | میں زیادہ نشاستےوالی خوراک (روٹی، چاول، آلو، وغیرہ) سےخصوصاً اجتناب کرتا/ کرتی ہوں۔ |  |  |  |  |  |  |
| 8 | میں محسوس کرتا/ کرتی ہوں کہ دوسرے لوگ چاہتے ہیں کہ میں زیادہ کھاؤں۔ |  |  |  |  |  |  |
| 9 | میں کھانے کے بعد قے کر دیتا/دیتی ہوں۔ |  |  |  |  |  |  |
| 10 | کھانے کے بعدمجھے شدیداحساسِ جرم ہوتا ہے۔ |  |  |  |  |  |  |
| 11 | مجھ پر دُبلا ہونے کی خواہش حاوی رہتی ہے۔ |  |  |  |  |  |  |
| 12 | ورزش کے دوران میں حراروں (Calories) کوکم کرنے کا سوچتا/سوچتی ہوں۔ |  |  |  |  |  |  |
| 13 | دوسرے لوگ یہ سوچتے ہیں کہ میں بہت دُبلا / دُبلی ہوں۔ |  |  |  |  |  |  |
| 14 | مجھ پر اپنے جسم کے موٹا ہو جانے کی سوچ حاوی رہتی ہے۔ |  |  |  |  |  |  |
| 15 | مجھے کھاناکھانے میں دوسروں سے زیادہ وقت لگتا ہے۔ |  |  |  |  |  |  |
| 16 | میں ان کھانوں سے اجتناب کرتا / کرتی ہوں جن میں چینی ہوتی ہے۔ |  |  |  |  |  |  |
| 17 | میں کم حراروں (Diet) والی غذا کھاتا/کھاتی ہوں۔ |  |  |  |  |  |  |
| 18 | مجھے لگتا ہے کہ جیسے کھانے نے میری زندگی پر قابو کیا ہوا ہے۔ |  |  |  |  |  |  |
| 19 | مجھے کھانے کی موجودگی میں خود پر قابورکھتا / رکھتی ہوں۔ |  |  |  |  |  |  |
| 20 | مجھے محسوس ہوتا ہے کہ دوسرے لوگ کھانے کےلئے مجھ پر دباؤ ڈالتے ہیں۔ |  |  |  |  |  |  |
| 21 | میں کھانے کو بہت وقت دیتا / دیتی اور سوچتا / سوچتی ہوں۔ |  |  |  |  |  |  |
| 22 | میں میٹھا کھانے کے بعد بے چینی محسوس کرتا/کرتی ہوں۔ |  |  |  |  |  |  |
| 23 | میں نے ڈائیٹنگ کے طور طریقے اپنائے ہیں۔ |  |  |  |  |  |  |
| 24 | مجھے خالی پیٹ رہنا پسند ہے۔ |  |  |  |  |  |  |
| 25 | کھانے کے بعد میری خواہش ہوتی ہے کہ قے کر دوں۔ |  |  |  |  |  |  |
| 26 | مجھے نِت نئے مرغن کھانوں کو کھانے میں لطف آتا ہے۔ |  |  |  |  |  |  |

| **حصہ سوئم: رویے سے متعلق سوالات**  **پچھلے چھے مہینوں کے دوران:** | | **کبھی نہیں** | **مہینے میں ایک دفعہ یا کم** | **مہینے میں 2-3 دفعہ** | **ہفتے میں 1 دفعہ** | **ہفتے میں 2-6 دفعہ** | **دن میں 1 دفعہ یا زیادہ** |
| --- | --- | --- | --- | --- | --- | --- | --- |
| **الف** | کیا آپ پر کھانے کا ایسا جنون سوار ہوا ہے کہ آپکو لگا ہو کہ آپ اپنے آپ کو روک نہیں سکتے ؟ |  |  |  |  |  |  |
| **ب** | کیا آپ نے وزن کم کرنے یا جسمانی خد و خال کو بہتر بنانے کے لیے قے کی ہے؟ |  |  |  |  |  |  |
| **ج** | کیا آپ نے وزن کم کرنے یا جسم کو خوبصورت بنانے کے لئے دست آور (Laxatives)، پیشاب آور (Diuretics) استعمال کی ہیں؟ |  |  |  |  |  |  |
| **د** | کیا آُپ نے اپنا وزن کم کرنے یا اُسکو بر قرار رکھنے کے لیے 60 منٹ یا اُُس سے زیادہ کی ورزش کی ہے؟ |  |  |  |  |  |  |
| **ہ** | کیا آُپ نےپچھلے 6 ماہ میں 20 پاؤنڈ یا زیادہ وزن گھٹایا ہے؟ | ہاں | | | نہیں | | |
